# The SPECTRA Study: Validating a New Memory Training Program based on the Episodic Specificity Induction to Promote Transfer in Older Adults

**DOI:** 10.5334/joc.323

**Published:** 2023-10-06

**Authors:** Rudy Purkart, Preslava Aleksieva, Samira Mellah, Gloria Leblond-Baccichet, Sylvie Belleville

**Affiliations:** 1 Centre de Recherche de l’Institut Universitaire de Gériatrie de Montréal, Montreal, CA; 2 Université de Montréal, Montreal, CA

**Keywords:** episodic specificity induction, cognitive training, transfer, aging, episodic memory

## Abstract

Some complex cognitive activities impacted by aging (future thinking, problem-solving, creative thinking) have been shown to rely on episodic retrieval, suggesting that cognitive interventions aiming to improve retrieval have the potential to induce transfer effects to these activities. Prior studies have shown that a brief one-session technique called Episodic Specificity Induction (ESI) can transiently improve episodic retrieval and induce transfer effects to complex tasks that rely on episodic retrieval in older adults. In the present proof-of-concept study, we assessed whether a training program consisting of repeated practice of the ESI technique can improve episodic retrieval and transfer to complex tasks. Fifteen healthy older adults completed a six-session intervention where they received repeated ESI practice. Before and after the intervention, nearest transfer effects were assessed using free recall, near transfer effects using recognition and associative recognition, and far-transfer effects using mean-ends problem-solving and divergent creative thinking. Before the intervention, typical ESI effects were observed (better performance after an ESI than after a control task), indicating that the ESI operated as expected in our sample. When examining the intervention effects, performance was increased after the intervention on free recall and recognition (nearest- and near-transfer) as well as problem-solving and divergent creative thinking (far transfer). These results indicate that an intervention relying on the ESI technique can produce both near and far transfer. These findings support the use of the ESI in the design of interventions that could improve retrieval and have a broad impact on a range of complex tasks.

## Introduction

Aging is associated with difficulties in episodic retrieval (Levine et al., 2002, see also Grilli & Sheldon, 2022, Korkki et al., 2020; Stark et al., 2013; Toner et al., 2009; Yassa et al., 2011), which is defined as the conscious recollection of sensory and contextual details associated with past events (Rugg & Wilding, 2000; see also Cabeza et al., 2000). Episodic retrieval is involved in the flexible recombination of event details that occur when imagining an event (see the constructive episodic simulation hypothesis, Schacter, 2022; Schacter et al., 2007; Schacter & Addis, 2007a, 2007b, 2020). As such, age-related difficulties in episodic retrieval may impact a broad range of cognitive tasks that involve imagining events, such as social problem-solving (e.g., Sheldon et al., 2011; Vandermorris et al., 2013) and divergent creative thinking (e.g., Reese et al., 2001; but for alternative observations, see Adnan et al., 2019; Palmiero et al., 2014). This suggests that improving episodic retrieval with cognitive intervention programs in older adults may have a positive impact on these complex cognitive abilities.

Cognitive training interventions are increasingly recognized as a relevant approach to reduce cognitive decline in older adults (National Academies of Sciences, Engineering, and Medicine et al., 2017), and a distinction is typically made between *strategy-based* and *process-based* interventions. A *strategy-based* intervention involves learning how and when to apply new or alternative methods for performing a particular cognitive task and thus heavily rely on metacognitive capacities. The aim of a strategy-based intervention is to compensate for the limitations of impaired processes by relying on those that are still intact. Training programs targeting episodic memory are most often *strategy-based* and typically teach mnemonics that are used to support rich and distinctive encoding (e.g., method of loci, peg words, mental imagery). They are designed to be used on a type of material that lends itself, and for this reason, are not meant to be generalized to other cognitive tasks or domains (Bahar-Fuchs et al., 2013, 2019; Belleville et al., 2006; Bottiroli et al., 2013; Hampstead et al., 2017; Neely & Bäckman, 1995; Strickland-Hughes & West, 2022; Yesavage, 1984).

In turn, a *process-based* intervention involves improving a particular cognitive ability by repeatedly exercising the underlying core cognitive process (for a review, see Willis & Belleville, 2016). The rationale is that it should produce gains that *transfer* to untrained cognitive abilities that rely on the same core process (von Bastian et al., 2022; Zimmermann et al., 2016). Based on the common element hypothesis (Woodworth and Thorndike, 1901; see also Noack et al., 2009; Sala & Gobet, 2017b, 2017a, 2019, 2020; Thorndike, 2013), transfer is often conceptualized as a continuum, depending on the extent to which the processes involved in the transfer task overlap with those targeted by the training task. *Nearest transfer* and *near transfer* tasks measure the same cognitive domain (e.g., memory) as the training task and involve a significant overlap in terms of the underlying processes. However, the distinction lies in the fact that near transfer tasks use different types of stimuli or a different task structure than nearest transfer tasks (e.g. recall vs. recognition). *Far transfer* measures are hypothesized to partly rely on the trained processes, otherwise no transfer would be expected to occur. However, it measures a different cognitive domain (e.g., memory vs. creativity). It is important to note that transfer tasks exist on a continuum of proximity to the trained task, and thus, determining a clear boundary between nearest, near, and far transfer measures remains somewhat arbitrary (see also Barnett and Ceci, 2002).

Therefore, one of the powerful benefits of process-based interventions is the possibility of producing far transfer effects. Surprisingly, very few training programs target episodic memory using a process-based approach, wich involves repeated practice of a task designed to target an underlying core process. Some of these programs have shown transfer effects. For example, it was shown that an associative memory training program that targeted the ability to encode and retrieve associations from long-term memory through the repeated practice of cued recall for object-location associations produced far transfer to reasoning (Zimmermann et al., 2016). This is because the ability to form stable associations would support the construction and manipulation of new structural representations required for reasoning. However, not all process-based memory interventions have led to transfer. For instance, another associative memory training study showed no transfer effect to reasoning (Bellander et al., 2017). Therefore, the reasons that explain the presence or absence of transfer following process-based memory interventions remain to be better understood.

The Episodic Specificity Induction (ESI) technique (Madore et al., 2014; for a short review, see Schacter & Madore, 2016) is a promising approach for a process-based memory training program that could lead to far transfer by improving episodic retrieval in older adults. The technique involves interviewing participants on their memory of an event (e.g., a videoclip) prior to the task of interest. It is based on a forensic interview protocol known to improve eyewitness testimony by promoting mental imagery and focusing recall on key aspects of an event (Cognitive Interview; Fisher & Geiselman, 1992; for a review, see Memon et al., 2010). During the interview, the interviewer focuses the participants’ retrieval attempts on specific aspects of the event: the scene (e.g., the objects, what they looked like, where they were located), people (e.g., what they looked like, where they were located) and actions (e.g., what was done, when and how). This is done by asking participants to mentally reinstate the details of each aspect (a technique known as *mental reinstatement)* and then report every detail, including those that do not seem important (a technique known as *integral report*).

According to the authors who developed the ESI technique, its effect would induce a *retrieval orientation* bias (Morcom & Rugg, 2012, see also Herron, 2018; Herron & Rugg, 2003). This bias would direct cognition toward retrieving key event-specific details (see the Readiness to Remember framework; Madore & Wagner, 2022) through the adoption and maintenance of an extended mnemonic goal state (see Schacter & Madore, 2016). This would improve the *event construction* process corresponding to the process of assembling and maintaining a coherent mental scene (Romero & Moscovitch, 2012). Once the ESI technique is administered, its effects are measured on a subsequent task of interest, and compared to a control task that does not target episodic retrieval. Two observations are of particular importance: First, the effects of the ESI technique extend for several minutes, which improves performance in a subsequent episodic memory task of interest. But most importantly, its positive effects extend to other complex cognitive tasks that also rely on episodic retrieval. Indeed, empirical evidence has shown that the prior administration of the ESI technique improved autobiographical recall in younger and older adults, but also improved tasks targeting episodic future thinking, divergent creative thinking and problem-solving (for a review, see Schacter & Madore, 2016). These improvements have been observed, even though the technique does not involve explicit teaching of a retrieval strategy for later use and was not reused by participants as a strategy in subsequent tasks. This suggests that the technique does not rely on metacognition but instead targets and improves a core episodic retrieval process involved in the tasks where the benefits of the technique have been observed, which should facilitate its use and applicability.

While the ESI technique has tremendous potential to improve complex tasks that rely on episodic retrieval in older adults, it has been typically provided and assessed in a single session, producing only transient improvements, and has never been adapted and tested as a memory training program. At this point, nothing is known about whether a training program consisting of repeated practice of the ESI technique can produce lasting improvements and transfer to complex tasks. Thus, one important step is to assess whether such a training program can improve episodic retrieval and induces transfer to complex tasks in older adults. The SPECTRA study (SPEcificity TRAining TRAnsfer) was specifically designed to address this aim.

## The present study

In the first phase of the SPECTRA study, we developed and co-designed with a focus group of older adults a memory training program that involved the repeated practice of the ESI technique (Purkart et al., submitted). The training program included six sessions, where the ESI technique was practised under guided supervision. Additionally, participants completed unsupervised homework involving self-administration of the ESI technique between sessions. To ensure the effectiveness of new non-pharmacological interventions, it has been proposed that a proof-of-concept study be conducted first, before proceeding to a costly and demanding randomized controlled trial (see The Obesity-Related Behavioral Intervention Trials (ORBIT) model; Czajkowski et al., 2015). As such, the present study corresponds to the second phase of the SPECTRA study, aimed at performing a proof of concept. According to the ORBIT model, ideal proof-of-concept studies employ quasi-experimental, treatment-only, and within-participant designs, where a small number of participants act as their own controls in a pre-post treatment comparison. With these recommendations in mind, the present study had three main objectives: First, we assessed whether the ESI technique operated as expected in a sample of 15 healthy older adults. This was done by comparing performance after the administration of an ESI technique (ESI condition) and after a control task (No-ESI condition) on tasks that had previously shown induction effects in other studies: free recall (Purkart, Versace, et al., 2019), social problem-solving (Madore & Schacter, 2014), and creative divergent thinking (Madore et al., 2016). This comparison was done at baseline, before participants began the ESI training program, to match the conditions of previous studies and compare findings. We also explored the effects of the prior administration of an ESI technique on recognition, as non-generative recognition tasks have rarely been used to assess induction effects. Including these variables and conditions was considered necessary to ensure that the desired processes are targeted effectively, and that the repeated practice of the ESI technique in a training program also targets these processes. It was expected that performance would be better in the ESI condition compared to the no-ESI condition. Second, the effect of the training program was evaluated by comparing participants’ baseline performance with their post-intervention performance, successively measured once again in the ESI condition and in the no-ESI condition. This effect was examined on free recall as a nearest-transfer outcome, and on problem-solving and creative divergent thinking as far-transfer outcomes, as induction effects were demonstrated in those tasks. We also explored intervention effects on recognition and associative recognition as near-transfer outcomes to test whether intervention effects were observable on accuracy, and influenced by the perceptive distinctiveness of old and new associations. It was expected that performance would improve for nearest, near and far transfer outcomes. This effect should be larger in the no-ESI condition because participants in the ESI condition should already benefit from the prior administration of an ESI technique. If there is a larger effect in the no-ESI condition, the induction effects found before the intervention (i.e., better performance in the ESI than in the no-ESI condition) should no longer be apparent after the intervention. Finally, retention was also evaluated by assessing how many participants remained in the study throughout its entire duration.

## Method

### Design

**Figure 1:**
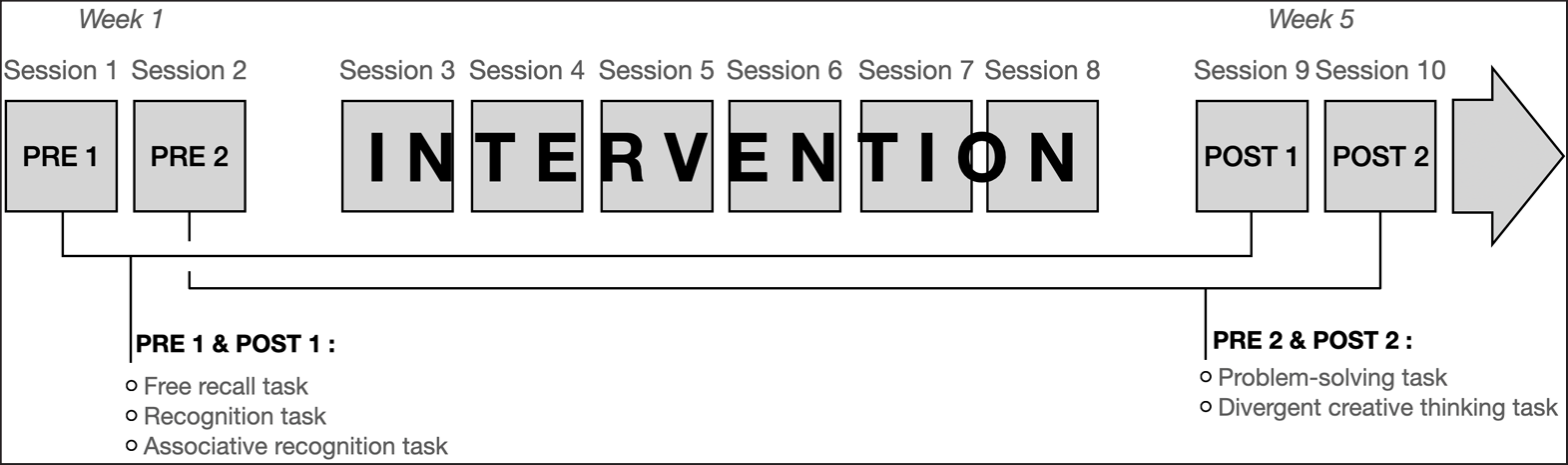
Schema of the study design following its co-creation.

The study was a single-arm pre-post intervention study with a focus on within-subject changes. The protocol adheres to the Consolidated Standards of Reporting statement (Begg et al., 1996). A schema of the study design is provided in Figure 1. There were two pre-intervention assessment sessions (PRE1 and PRE2, sessions 1 and 2), six training sessions (sessions 3, 4, 5, 6, 7 and 8), and two post-intervention assessment sessions (POST1 and POST2, sessions 9 and 10). PRE1 was identical to POST1 except for the material used, and this was also the case for PRE2 and POST2. All sessions lasted two hours and were provided twice per week. PRE1 and POST1 assessment sessions included three episodic memory tasks and a clinical assessment (only in the PRE1 assessment session). PRE2 and POST2 assessment sessions included the problem-solving and divergent creative thinking tasks. Assessment sessions took place in a quiet testing room in the research center. Training sessions were provided in small groups of four individuals and took place in a group intervention room in the research center. The research conformed with the ethical rules for human experimentation stated in the Declaration of Helsinki and was approved by the Comite d’éthique de la recherche vieillissement et neuroimagerie of the Centre intégré universitaire de santé et de services sociaux (CIUSSS) du Centre-Sud-de-l’Île-de-Montréal (CER VN 21-22-28).

### Participants

Fifteen community-dwelling cognitively intact older adults (M_age_ = 75.7 years; SD_age_ = 5.93 years; 2 males) were recruited from the participant registry of the Centre de recherche de l’Institut universitaire de gériatrie de Montréal (CRIUGM) in Montreal, Canada. Participants were included if they were fluent in French, had corrected or sufficient visual and auditory acuity to undergo neuropsychological testing, obtained a score equal or superior to 26 on the Montreal Cognitive Assessment (MoCA; Nasreddine et al., 2005), and performed above the education-adjusted cut-offs on the delayed recall portion of the Logical Memory Test of the Wechsler Memory Scale for older adults used in the Alzheimer’s Disease Neuroimaging Initiative (ADNI) study (Chapman et al., 2016; Elwood, 1991; Petersen et al., 2001). The mean years of education was 16 (*SD* = 3.63), the mean MoCA score was 27.5 (*SD* = 1.68), and the mean score on the delayed recall portion of the Logical Memory Test was 16.3 (*SD* = 4.54).

Participants were excluded if they had a central nervous system disease diagnosis or injury, multiple sclerosis, neurodevelopmental disorders, subdural hematoma, subarachnoid hemorrhage, primary cerebral tumor or cerebral metastases, epilepsy, neurodegenerative diseases, stroke, major surgery within the last two months, substance abuse, general anesthesia in the past six months, serious co-morbid conditions, major depression or anxiety, or major psychiatric disorders. Participants received financial compensation for their participation.

### Intervention

The goal of the intervention was to practice the ESI technique with different materials and instructions. Training sessions were conducted in groups of 4 participants and were led by an experimenter. The first three training sessions began with a short psychoeducation component aimed at increasing engagement and motivation. This component provided participants with knowledge about episodic memory and the specific targets of the program. Importantly, memory strategies were not discussed during psychoeducation. Subsequently, participants practiced a different version of the ESI technique per session. In the first training session, participants encoded four different videoclips of a complex scenario featuring the famous British comic character Mr. Bean performing common activities in a familiar location (e.g., ordering food in a restaurant). Each participant was then interviewed about their memory of one of the four videoclips using the standard version of the ESI technique (Madore et al., 2014). In the second training session, participants selected a place, person, and object related to a specific autobiographical memory and were interviewed about this memory using the autobiographical version of the ESI technique (Madore et al., 2018). In the third training session, participants selected a place, person and object related to different autobiographical memories, and were asked to imagine a future event based on these details using the imagination version of the ESI technique (Madore et al., 2018). It is worth noting that the three versions of the ESI technique produced a similar increase in performance on a free recall task and are assumed to target a same core retrieval process (Madore et al., 2014, 2018). All three versions are based on the techniques of the cognitive interview and are very similar, except for the type of memories targeted (video memories, autobiographical memories) and the temporal orientation (past, future). The last three training sessions were identical to the first training session, except that the psychoeducation component was replaced by illustrations demonstrating how to use each ESI version in everyday life. Between training sessions, participants were asked to complete homework, totalling six homework assignments per participant by the end on the intervention. Each homework assignment was expected to take about 15–30 minutes to complete and involved practicing the version of the ESI technique that was taught during the last training session, using a booklet containing self-administration instructions. All participants who completed the POST-intervention assessment completed all their homework. Instructions for the next training session’s homework were provided and explained at the end of each session, and completed homework was reviewed and discussed at the beginning of each subsequent training session. The homework was used primarily to provide participants with additional practice of the ESI technique and the opportunity to continue using it unsupervised after the study.

#### Outcome measures

**Figure 2:**
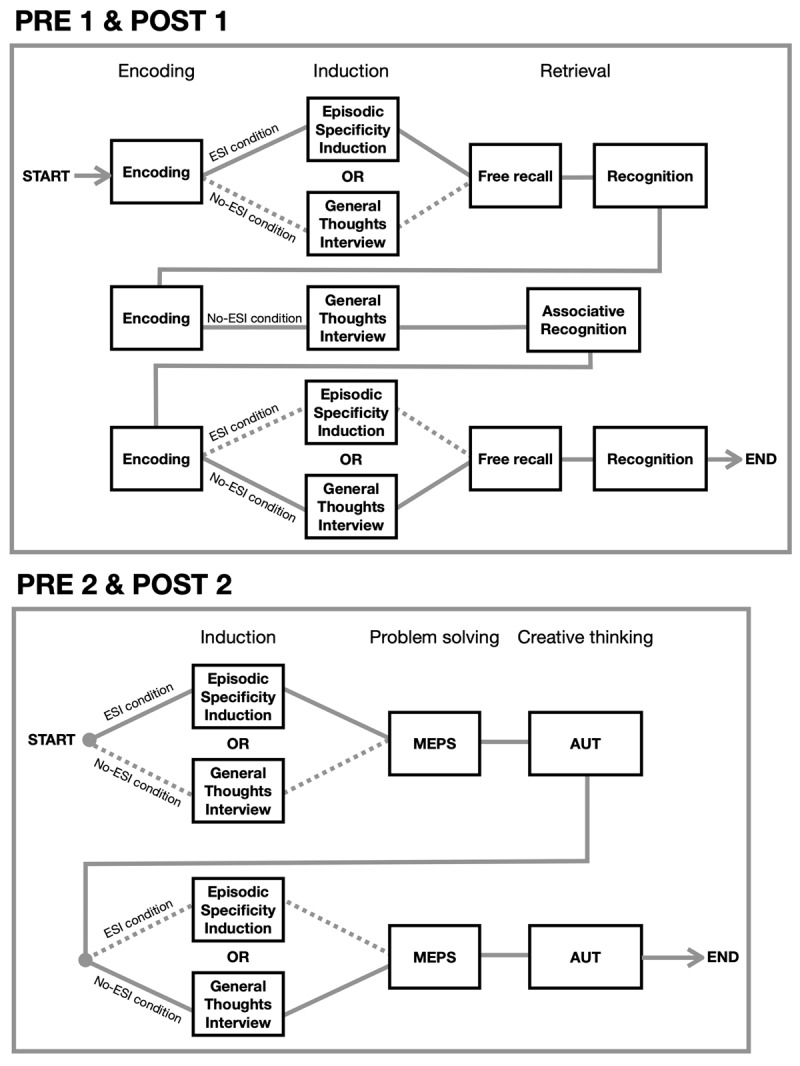
Order of the tasks. **PRE1 & POST1:** each of the three successive segments started with an encoding phase, followed by an induction phase where an ESI technique (ESI condition) or a control task (No-ESI condition) was administered in a counterbalanced order (except for Segment 2, where only the control task was administered). Each segment concluded with a retrieval phase, where the free recall and recognition tasks were administered in a counterbalanced order (except for Segment 2, where only the associative recognition task was *administered*). **PRE2 & POST2:** each of the two successive segments started with an induction phase, where an ESI technique (ESI condition) or a control task (No-ESI condition) was administered in a counterbalanced order, followed by the problem-solving task (MEPS) and the divergent creative thinking task (AUT), administered in a counterbalanced order. *Note*: MEPS = Means-End Problem Solving task, AUT = Alternate Uses Task, ESI = Episodic Specificity Induction.

**Figure 3:**
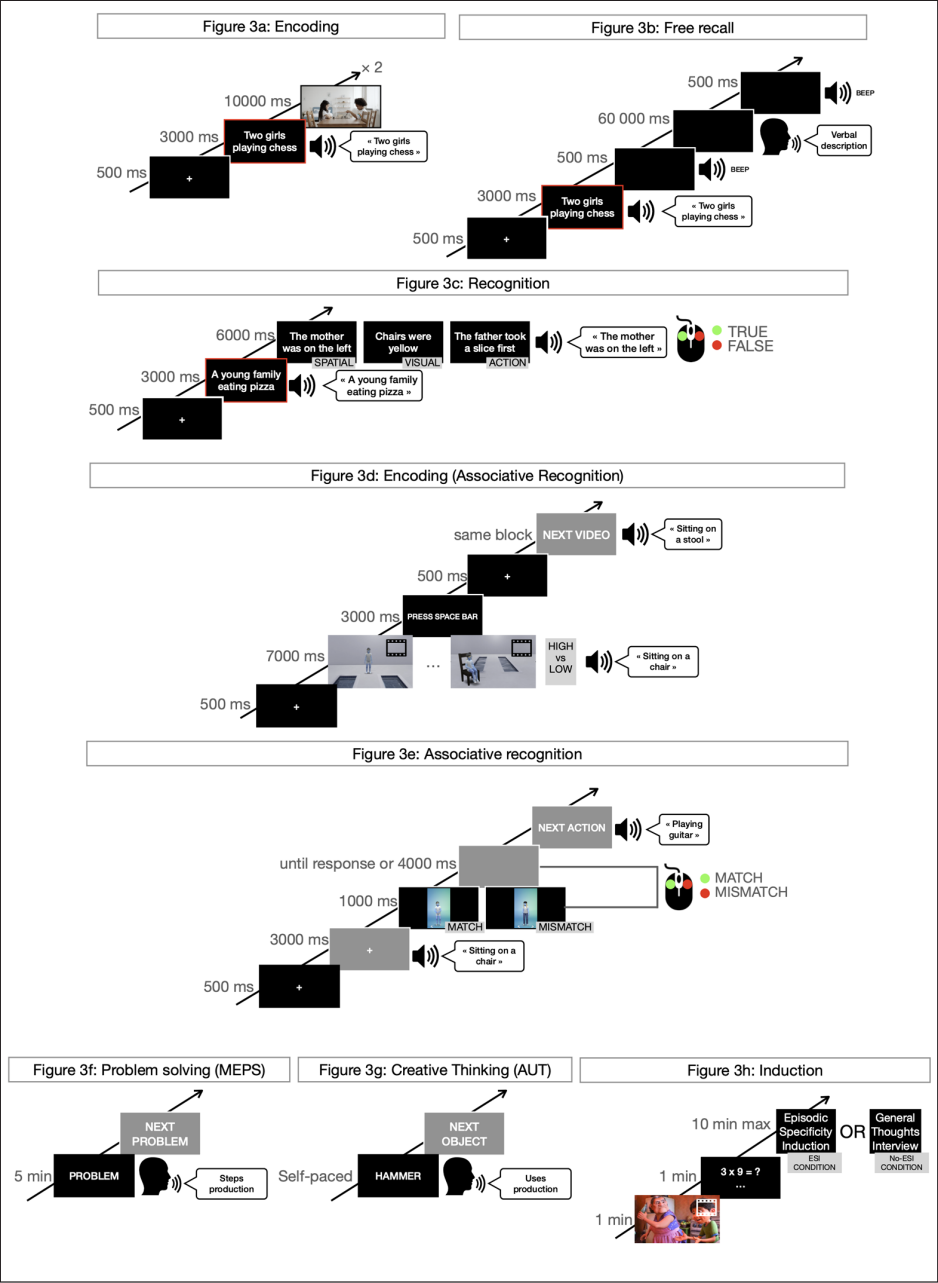
Examples of one trial in each task. **3a:** A video title was presented visually and auditorily, immediately followed by the corresponding video (This sequence was replicated twice for each video.). **3b:** Participants were prompted with a video title and required to verbally describe their memory of the corresponding video in as much detail as possible. **3c:** Following the display of a video title, a statement about a spatial, visual or actional aspect of the corresponding video was visually and auditorily presented. Participants were then tasked with determining the statement’s veracity using a mouse-based response. **3d:** An audio description accompanied a video depicting a character performing an action on an object. Subsequently, the next three videos showcased characters from the same block, doing the same action on a different object, and originating either from the high or low distinctiveness condition. **3e:** An audio description of a specific action on a given object was presented, immediately followed by an image of a character that may or may not match the one who executed the action. Participants were instructed to assess whether the character matched or not, using the mouse. **3f:** Participants were asked to verbally describe the steps that led a protagonist – or themselves – from the beginning of a social problem to its resolution. **3g:** In response to an object cue, participants were asked to verbally describe as many creative and alternative uses as possible. **3h:** After watching a video and completing math exercises, participants were either interviewed about their memory of the video using Episodic Specificity Induction (ESI condition) or asked to share their general thoughts about the video (No-ESI condition). *Note*: MEPS = Means-End Problem Solving task, AUT = Alternate Uses Task, ESI = Episodic Specificity Induction.

The tasks were programmed using the free software OpenSesame (Version 3.3.14; Mathôt et al., 2012). Instructions and stimuli were presented on an external computer monitor, and responses were collected with a mouse or keyboard depending on the task. For each participant and assessment session (PRE- and POST-intervention), the four outcome measures were assessed under both the ESI and no-ESI conditions. The order of tasks, conditions (ESI condition first vs. last), and the material used (e.g., videos) were counterbalanced. The order of the tasks is presented in Figure 2, and an example of one trial for each task is shown in Figure 3. Further details about the material is provided in the Supplementary Material.

#### Nearest and near transfer tasks

##### Free recall and recognition

The free recall task was adapted from Purkart, Versace, et al. (2019) and was used to assess nearest transfer. The recognition task was inspired by Sheldon et al. (2017) and was used to assess near transfer. The free recall and recognition tasks shared and started with the same encoding phase, where participants were shown a fixation cross (500 ms) and a video title (e.g., “Two little girls playing chess”), which was presented visually and auditorily (3000 ms), followed by the presentation of a 10-second video. The video depicted a scenario involving characters in a real-world indoor environment that was rich in objects, furniture, characters, and action. Participants were instructed to pay attention and memorize as many details as possible. This sequence was repeated twice for each of the 10 different videos presented.

The encoding of the 10 videos was followed by an induction phase where participants were presented a one-minute videoclip depicting a scenario involving animated characters in a rich fictional environment. They were instructed to pay attention to both the general aspects and specific details of the video as they might later be asked about them. After a one-minute filler task (simple math exercises), participants in the ESI condition were interviewed using the standard ESI, while participants in the no-ESI condition were interviewed using the general thoughts interview (Madore et al., 2014). The standard ESI and the general thoughts interview used in the study were French adaptations (Purkart, Vallet, et al., 2019). In the standard ESI, participants were asked to recall three aspects of the video sequentially: the scene, characters and actions. For each aspect, they had to mentally reinstate the details related to the aspect (e.g., for the scene: the objects and furniture, and their appearance and location) with their eyes closed and describe all possible details of the aspect, even those that seemed irrelevant. In the general thoughts interview, participants were asked to share their general thoughts about the video in response to the examiner’s prompts (e.g., do you think the video is suited for children? Was it funny?). This interview was designed to avoid targeting retrieval of specific episodic details.

The retrieval phase immediately followed the induction phase, which was done with free recall for five of the videoclips and recognition for the other five. For free recall, participants were presented with a fixation cross (500 ms), followed the title of an encoded videoclip (3,000 ms), and were asked to recall as many details as possible about the corresponding videoclip within one minute. Participant responses were audio recorded, transcribed, and scored. Two independent and trained raters that were blind to the induction condition coded each transcript for *specific details* based on the Autobiographical Interview scoring manual (Levine et al., 2002). A specific^1^ detail is a part of a sentence that conveys unique information about a perceptual, spatial, temporal, emotional or actional aspect of the specific event recalled. Inter-rater reliability was assessed on a training packet of 20 responses where a high agreement was reached (Cronbach’s α = .88). A different blind rater verified their accuracy against the video clips. The dependent variable was the mean number of correct specific details recalled.

For recognition, participants were presented with a fixation cross (500 ms), followed by one of the five remaining titles of the encoded videoclips (3,000 ms). For each videoclip, six statements were successively presented both visually and auditorily. Two concerned visual aspects of the videoclips (e.g., “the chairs were yellow”), two concerned spatial aspects (e.g., “the mother was on the left”), and two concerned the actions carried out (e.g., “the father took a slice first”). For each aspect, one statement was true, and one was false. Participants had 6,000 ms to indicate if the statement was true or false using the corresponding mouse key. Hits, misses, false alarms and correct rejections were collected to compute a discriminability criterion (d’), which was calculated according to Swets (2014) and Wickens (2001):


1 \[d^{\prime} = z(hit\ rate) - z(false\ alarm\ rate)\]

##### Associative recognition

The associative recognition task was reproduced from Purkart et al. (2022) and used to assess near transfer. The task started with an encoding phase. Following a fixation cross (500 ms), a seven-second animated video portrayed a character performing an action on an object (e.g., sitting on a chair). During the videoclip, the action being performed, and the object’s name were presented auditorily. Participants were instructed to focus on and memorize the character, the action performed, and the object’s name. A total of six blocks, each containing four videoclips, were presented (24 in total). In half of the blocks, the characters within a block were perceptually similar (LOW DISTINCTIVENESS context), while in the other half, characters were dissimilar (HIGH DISTINCTIVENESS context). For additional details, see Purkart et al. (2022). Following the encoding of the 24 videoclips, an induction phase took place under the no-ESI condition. Due to the limited number of available videos, participants exclusively performed the task in the no-ESI condition to ensure an adequate number of trials. Subsequent to the induction phase, the retrieval phase promptly ensued. Participants were presented auditorily with the 24 actions and object names (lasting 3,000 ms), each followed by an image of a previously encoded character (lasting 1,000 ms). Participants had a 4,000 ms window to indicate via a mouse key press whether the character performed the given action or not. Hits, misses, false alarms, and correct rejections were recorded to compute the d’ criterion.

#### Far transfer tasks

##### Problem-solving

Problem-solving was immediately evaluated following the induction phase using the means-end problem-solving task (MEPS) (Platt & Spivack, 1975). Participants were presented with hypothetical social problems, along with solutions to those problems, and were asked to generate the steps that would lead to the solution in as much detail as possible within five minutes (e.g., “You would like to declutter your living space. The story ends with you decluttering your living space. The story begins with you wanting to declutter your living space.”). Two stories were introduced in the first person and presented a situation previously identified as self-relevant and problematic by an independent sample of older adults (Madore & Schacter, 2014). Two stories were introduced in the third person and selected from Platt & Spivack (1975). Participants were informed that they could move on to the next problem if they considered the problem solved. Participant responses were audio recorded, transcribed, and counted. The dependent variable was the mean number of relevant steps produced.

##### Divergent creative thinking

Divergent creative thinking was immediately evaluated following the means-end problem-solving task using the Alternate Uses Task (AUT) (Guilford, 1967). Participants were presented five common objects (e.g., newspaper, eyeglasses, umbrella) and asked to produce as many alternative and creative uses as possible for each item without time constraints. They were allowed to move on to the next object if they ran out of ideas. Participant responses were audio recorded, transcribed, and scored. The dependent variables were the mean number of appropriate use categories (e.g., using paper clips to make a necklace or a bracelet are two uses that belong in the same “jewelry” category) and mean creativity score (from 1 to 4 for each use, with 3 and 4 reserved for highly creative uses that would only be produced by a few people) (for the manual, see Guilford et al., 1960). Two independent raters, who were blind to the induction condition received, coded responses for the number of categories and creativity scores. Inter-rater reliability was assessed on a training packet of 40 responses for which an acceptable agreement was reached (Cronbach’s α = .84 for number of categories of appropriate uses; Cronbach’s α = .79 for creativity).

## Results

### Data analyses

Data were analyzed using R Studio 3 software (version 2022.07.1). Linear mixed-effects models were performed using the lmerTest R package (Kuznetsova et al., 2017). The Kenward–Roger degrees-of-freedom method was used for all analyses. Contrasts were performed using the emmeans R package (Version 1.6.0) and corrected with the Bonferroni method. The threshold of statistical significance for all analyses was set to *p* < .05.

Induction effects were investigated by performing linear mixed-effects models, comparing the ESI condition to the no-ESI condition at the PRE-intervention stage across the dependent variables of the free recall, recognition, problem-solving, and creative thinking tasks. For all the outcome measures, the fixed effect was the induction condition (ESI vs. no-ESI). The order in which participants performed the induction condition (Order: ESI condition first vs. last) was also included as fixed effect to assess whether receiving the ESI first influenced performance in the No-ESI condition. Participants were included as random effect. Induction effects were supported if performance was better in the ESI condition compared to the no-ESI condition.

Intervention effects were investigated by performing linear mixed-effects models for the nearest and near transfer outcome measures (free recall, recognition and associative recognition tasks), as well as far transfer outcome measures (MEPS and AUT tasks), focusing on the dependent variables of interest. For all the tasks except for the associative recognition task, the fixed effects comprised the induction condition (ESI vs. no-ESI), time (PRE vs. POST), and order (ESI condition first vs. last). For the associative recognition task, the fixed effects comprised the distinctiveness condition (LOW DISTINCTIVENESS vs. HIGH DISTINCTIVENESS) and time (PRE vs. POST). Participants were included as random effect. Intervention effects were supported if performance increased from PRE to POST, particularly in the no-ESI condition. Only one participant was unable to partake in the post-intervention assessment sessions due to unavailability.

The process of model selection involved a comparison of the goodness-of-fit between main effects models and those incorporating interactions among fixed effects. This was achieved using the *anova*() R function and likelihood ratio tests for nested models. Interaction models were selected when their associated p-values were below the statistical significance level. It should be noted that none of the models incorporating induction order as a fixed effect achieved significance and therefore were not chosen for any of the outcome measures or analyses. Detailed structures of the selected models are outlined in Appendix A, while descriptive statistics are reported in Table 1.

**Table 1:** Descriptive statistics.


TASKS	INDEPENDANT VARIABLES			DEPENDANT VARIABLES	
		
FREE RECALL		INDUCTION	TIME	CORRECT SPECIFIC DETAILS	

				M	SD

		No-ESI	PRE	13.32	2.89

			POST	22.88	3.18

		ESI	PRE	16.37	2.81

			POST	22.87	3.67

**RECOGNITION**	**STATEMENT**	**INDUCTION**	**TIME**	**d’**	

				**M**	**SD**

	Action	No-ESI	PRE	0.79	0.72

			POST	0.80	0.61

		ESI	PRE	0.64	0.64

			POST	0.84	0.88

	Spatial	No-ESI	PRE	0.52	0.41

			POST	1.27	0.60

		ESI	PRE	0.89	0.71

			POST	1.12	0.44

	Visual	No-ESI	PRE	0.53	0.78

			POST	0.60	0.68

		ESI	PRE	0.64	0.93

			POST	1.03	0.62

**ASSOCIATIVE RECOGNITION**	**DISTINCTIVENESS**		**TIME**	**d’**	

				**M**	**SD**

	HIGH		PRE	0.52	0.80

			POST	0.33	1.03

	LOW		PRE	0.22	0.98

			POST	0.12	0.55

**PROBLEM SOLVING (MEPS)**		**INDUCTION**	**TIME**	**RELEVANT STEPS**	

				**M**	**SD**

		No-ESI	PRE	4.86	3.12

			POST	9.89	4.63

		ESI	PRE	9.09	3.71

			POST	11.09	4.20

**DIVERGENT CREATIVE THINKING (AUT)**		**INDUCTION**	**TIME**	**CREATIVITY SCORE**	

				**M**	**SD**

		No-ESI	PRE	1.69	0.19

			POST	2.16	0.18

		ESI	PRE	1.87	0.18

			POST	2.29	0.19

		**INDUCTION**	**TIME**	**CATEGORIES OF APPROPRIATE USES**	

				**M**	**SD**

		No-ESI	PRE	18.43	4.18

			POST	19.50	4.24

		ESI	PRE	17.86	5.43

			POST	17.93	4.67


MEPS: Means-End Problem Solving Task; AUT: Alternate Uses Task (divergent creative thinking task).

### Induction effects

The analysis investigating induction effects in free recall at baseline indicated a larger mean number of correct specific details in the ESI condition than in the No-ESI condition at the PRE-intervention (*b* = 3.05, *SE* = 0.86, *t* = 3.53, *p* < .001, 95% CI [1.35, 4.74]). Analysis of recognition at baseline indicated no difference in the d’ criterion between the ESI condition and the no-ESI condition at the PRE-intervention for action (*b* = –0.14, *SE* = 0.22, *t* = –0.64, *p* = .53, 95% CI [–0.58, 0.30]), spatial (*b* = 0.37, *SE* = 0.18, *t* = 2.01, *p* = .06, 95% CI [-0.002, 0.74]) and visual statements (*b* = 0.11, *SE* = 0.31, *t* = 0.35, *p* = .72, 95% CI [-0.51, 0.73]).

Analysis of problem-solving (MEPS) at baseline indicated a larger mean number of relevant steps in the ESI condition than in the no-ESI condition at PRE-intervention (*b* = 3.75, *SE* = 0.97, *t* = 3.83, *p* < .01, 95% CI [1.77, 5.73]). Analysis of the divergent creative thinking task (AUT) at baseline indicated a higher creativity score in the ESI condition than in the no-ESI condition at the PRE-intervention (*b* = 0.20, *SE* = 0.06, *t* = 3.22, *p* < .01, 95% CI [0.08, 0.31]). However, the mean number of categories of appropriate uses was not significantly different between the ESI condition and the no-ESI condition at the PRE-intervention (*b* = –0.57, *SE* = 1.99, *t* = –0.29, *p* = .78, 95% CI [–4.60, 3.46]).

### Intervention effects

#### Nearest-transfer outcome measure

##### Free recall

**Figure 4:**
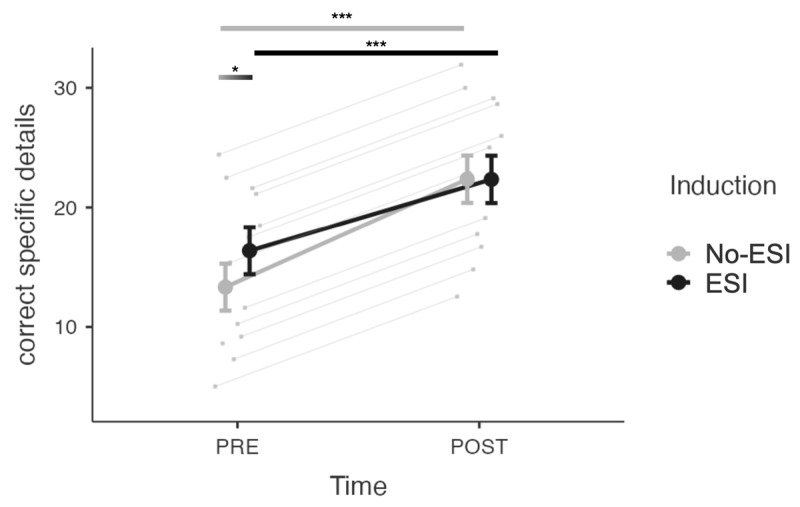
Mean number of correctly recalled specific details for free recall in the ESI and no-ESI conditions during the PRE- and POST-intervention assessments. *Note*: Bars represent standard errors. * *p* < .05, ** *p* < .01, *** *p* < .001.

For the analysis investigating intervention effects on free recall, the interaction model was selected over the main effects model. The analysis revealed no significant main effect of induction (*b* = –0.02, *SE* = 0.95, *t* = –0.02, *p* = .99, 95% CI [–1.96, 1.93]). However, a significant main effect of time (*b* = –9.04, *SE* = 0.99, *t = –*9.16, *p* < .001, 95% CI [–10.97, –7.11]) was observed, along with a significant induction × time interaction (*b* = 3.06, *SE* = 1.38, *t* = 2.26, *p* = .03, 95% CI [0.36, 5.76]). Contrast analyses revealed an increase in the mean number of correctly remembered specific details from the PRE to POST-intervention stages in both the no-ESI (*t*(235) = 9.16, *p* < .0001, *d* = 1.66) and ESI (*t*(235) = 6.06, *p* < .0001, *d* = 1.10) conditions. Interestingly, while a lower mean number of correctly recalled specific details was observed in the no-ESI condition compared to the ESI condition during the PRE-intervention assessment (*t*(234) = *–*3.18, *p* < .01, *d* = 0.56) (Figure 4), this discrepancy disappeared at the POST-intervention, where both conditions demonstrated equivalent mean numbers of correct specific details (*t*(234) = 0.02, *p* = .99, *d* = -0.003).

#### Near-transfer outcome measures

##### Recognition

In the analysis investigating intervention effects on the recognition task, main effects models were selected over interaction models for spatial, action and visual information. For the spatial information, the analysis revealed no significant main effect of induction (*b* = 0.12, *SE* = 0.14, *t* = 0.83, *p* = .41, 95% CI [–0.16, 0.40]) but a significant effect of time emerged (*b* = –0.48, *SE* = 0.14, *t = –*3.33, *p* < .01, 95% CI [–0.77, –0.20]), indicating a higher d’ criterion POST-intervention compared to PRE-intervention. For the action information, the analysis revealed no main effect of induction (*b* = –0.05, *SE* = 0.17, *t = –*0.32, *p* = .75, 95% CI [–0.39, 0.28]) or time (*b* = –0.09, *SE* = 0.17, *t = –*0.52, *p* = .61, 95% CI [–0.43, 0.25]). For the visual information, the analysis also revealed no main effect of induction (*b* = 0.27, *SE* = 0.20, *t* = 1.34, *p* = .18, 95% CI [–0.118, 0.65]) or time (*b* = –0.23, *SE* = 0.20, *t = –*1.149, *p* = .25, 95% CI [–0.61, 0.16]).

##### Associative recognition

For the analysis investigating intervention effects on the associative memory task, the main effects model was selected over the interaction model. The analysis indicated no significant main effect of distinctiveness (*b* = –0.25, *SE* = 0.23, *t* = –1.125, *p* = .27, 95% CI [–0.70, 0.19]) or time (*b* = 0.15, *SE* = 0.23, *t* = –0.68, *p* = .50, 95% CI [–0.30, 0.60]).

#### Far-transfer outcome measures

##### Problem-solving

**Figure 5:**
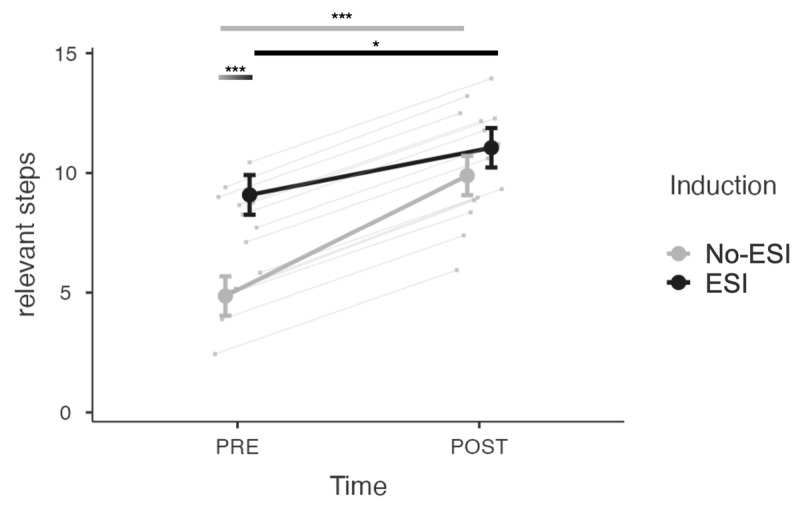
Mean number of relevant steps for the MEPS task in the ESI and no-ESI conditions at PRE- and POST-intervention. *Note*: Bars represent standard errors. *p < .05, **p < .01, ***p < .001.

For the analysis investigating intervention effects on problem-solving, the interaction model was selected over the main effects model. The analysis revealed no significant main effect of induction (*b* = 1.16, *SE* = 0.67, *t* = 1.730, *p* = 0.08, 95% CI [–0.15, 2.47]). However, a significant effect of time (*b* = –5.04, *SE* = 0.67, *t = –*7.54, *p* < .001, 95% CI [–6.34, –3.73]) and induction × time interaction was observed (*b* = 3.07, *SE* = 0.95, *t* = 3.22, *p* < .01, 95% CI [1.20, 4.93]). Contrast analyses indicated that there was a significant increase in the number of relevant steps from the PRE- to POST-intervention in both the no-ESI (*t*(203) = 7.54, *p* < .001, *d* = 1.42) and ESI (*t*(203) = 2.89, *p* < .01, *d* = 0.55) conditions. However, while there was a smaller mean number of relevant steps produced in the no-ESI compared to the ESI condition at the PRE-intervention (*t*(203) = *–*6.24, *p* < .001, *d* = 1.20)(Figure 5), this was no longer the case at the POST-intervention where both conditions were equivalent (*t*(203) = *–*1.73, *p* = .17, *d* = 0.33).

##### Divergent creative thinking

For the analyses investigating intervention effects on the mean number of categories and the creativity score, main effects models were selected over interaction models. The analysis on the mean number of categories revealed no main effect of induction (*b* = –1.07, *SE* = 1.23, *t* = –0.97, *p* = .40, 95% CI [–3.48, 1.34]), nor was there a significant effect of time (*b* = –0.57, *SE* = 1.23, *t* = –0.46, *p* = .64, 95% CI [–2.98, 1.84]). The analysis on the creativity score revealed a significant main effect of induction (*b* = 0.15, *SE* = 0.04, *t* = 3.38, *p* < .001, 95% CI [0.06, 0.24]), indicating a higher creativity score in the ESI condition compared to the no-ESI condition. Additionally a significant main effect of time was observed (*b* = –0.43, *SE* = 0.045, *t = –*9.46, *p* < .001, 95% CI [–0.52, –0.34]), signifying a higher creativity score at the POST-intervention stage.

## Discussion

This proof-of-concept study aimed to assess whether a training program consisting of repeated practice of the ESI technique can improve a core episodic retrieval process and induce transfer to complex tasks that rely on this process in healthy older adults. First, we evaluated whether the ESI technique used here operated as expected in this sample of older adults by assessing induction effects before the intervention. We expected that induction effects would be observed through better performances after an ESI technique (ESI condition) than after a general thoughts interview (No-ESI condition). We then assessed whether the intervention increased performance on nearest, near and far transfer outcome measures by comparing performance before and after the intervention in the ESI and no-ESI conditions. Finally, we also evaluated participant retention by assessing how many participants remained during the entire study. Each of these broad goals are discussed below.

### Induction effects

As a first step, we assessed whether the expected induction effect would be observed as reported in literature. This was important because it would be unlikely for an intervention effect to be observed if our induction conditions did not yield the induction effect reported in literature. This effect was examined at baseline on three tasks (free recall, problem-solving and divergent creative thinking) that have relatively solid evidence for the presence of an induction effect.

We observed the expected induction effect on free recall: administering ESI just prior to the task increased the number of correct specific details recalled. This finding extends the induction effects previously found in younger adults on this task to older adults (Purkart, Versace, et al., 2019), and is consistent with the induction effects found in younger and older adults in autobiographical recall (Madore et al., 2014, 2015, 2018; Madore & Schacter, 2014). Note that in contrast to prior studies, we evaluated whether the specific details produced were correct. This allowed us to assess whether the increase in performance induced by the prior administration of an ESI simply reflected an increase in the amount of information produced or an improvement in the accuracy of the information retrieved. This result is important because it indicates that the ESI technique improves episodic retrieval precision rather than simply increasing verbal output at the expense of accuracy.

We also observed the expected induction effects on problem-solving and creative thinking. For problem-solving, administering an ESI technique just prior to the task increased the number of relevant steps produced, thus replicating previous studies (Madore & Schacter, 2014). Regarding creative thinking, an induction effect was observed on the creativity score but not on the number of categories of appropriate uses. This differs from a prior study, which reported the opposite pattern in younger and older adults (Madore et al., 2016). It is possible that participants in the two studies differed in their emphasis of the creativity instruction (i.e., “focus on generating creative and unusual uses”) or fluency instruction (i.e., “list as many other uses for the object cue as you can”) (see Nusbaum et al., 2014).

In an exploratory manner, we examined induction effects on recognition. This was motivated by the lack of evidence regarding ESI effects on non-generative tasks, such as recognition. No induction effects were observed on the discriminability index for any of the statement types. However, as contradictory results have been reported (see Purkart et al., 2022), the issue deserves more investigation.

Overall, these results replicate or extend to older adults the induction effects found in previous studies, which confirms that the ESI technique used here operated as expected. These results also provide additional evidence that the ESI technique targets and improves core processes involved in the abilities we assessed. According to the constructive episodic simulation hypothesis (Schacter & Addis, 2007a, 2007b), remembering, problem-solving and divergent creative thinking are hypothesized to involve the retrieval of event-specific and previously experienced details and their recombination during the process of *scene construction* (Hassabis & Maguire, 2007) or *event construction* (Romero & Moscovitch, 2012). Administering an ESI technique is hypothesized to induce a bias of *retrieval orientation* (i.e., the process of engaging a specific form of processing on a retrieval cue; Herron & Rugg, 2003; Morcom & Rugg, 2012; Rugg & Wilding, 2000) toward key event-specific details (Schacter & Madore, 2016). This bias would help assemble a coherent mental scene by filling it with key event-specific details.

### Intervention effects

Our second goal was to assess whether a training program, comprising repeated practice of the ESI technique, could improve episodic retrieval and induce transfer to complex tasks reliant upon it among healthy older adults. Free recall was used as a nearest-transfer measure, while recognition and associative memory were used as near-transfer measures. Furthermore, problem-solving and creative thinking were used as far-transfer measures.

We observed interesting intervention effects for the nearest transfer outcome measure: The intervention increased the number of correct specific details recalled in the free recall task in both the ESI and no-ESI conditions. Interestingly, the intervention also increased the discriminability index for spatial statements in the recognition task (near transfer). Previous work has shown an impact of the prior administration of an ESI technique on metrics of scene construction underlying the spatial aspects of a mental scenario (Madore et al., 2019). As the intervention consisted in the repeated practice of the technique, the core processes targeted and improved by the prior administration of the technique might have been further improved by the intervention. Accordingly, the intervention may have improved the assembly of a spatially-coherent scene during scene construction, which facilitated the recall of correct specific details during free recall, and the discrimination of spatial statements during recognition. Our result on recognition is interesting because no induction effects were found at baseline. It is therefore possible that the prior administration of an ESI technique produced a slight increase in discrimination of spatial statements at baseline, but the small sample size did not provide enough power to make this effect significant, whereas repeated practice of the technique produced a larger increase.

It should be noted that an intervention effect was not observed on the associative recognition task, which was used as an exploratory near-transfer task. Therefore, any conclusions pertaining to the near-transfer effect of the intervention should be taken with caution. One prior study reported an induction effect on this task in the low distinctiveness condition in younger adults (Purkart et al., 2022). However, in the present case, the task may have been too difficult for older adults to benefit from the intervention. This interpretation is supported by a low discriminability index in the low distinctiveness condition. Another noteworthy aspect is our utilization of video game design, a format that older adults may be less familiar with, potentially contributing to their low discrimination sensitivity overall.

Importantly, we also observed the expected transfer effects to far transfer outcome measures: The intervention led to an increase in the number of relevant steps generated in the problem-solving task, as well as an increased creativity score in the creative thinking task. Given the presence of induction effects within these tasks, it is plausible that the core processes targeted and improved by the prior administration of an ESI technique might have experienced further enhancement due to the intervention. This improvement, in turn, contributed to enhanced performance across tasks reliant on the aforementioned core processes. These results are encouraging because they suggest that the intervention produced transfer to complex cognitive tasks.

It is worth noting that our analyses revealed that, during the POST-intervention assessment session, receiving an ESI technique immediately before the free recall and problem-solving tasks did not yield any additional benefits compared to the intervention effects alone. In other words, receiving the technique immediately before the tasks did not provide additional benefits, given that the technique had already been extensively practiced during the intervention. This may be due to the fact that after the intervention, participants’ performance reached an asymptote and could not improve any further with additional sessions (or doses) – an effect observed in other intervention studies (Belleville et al., 2022). Finally, participant retention was very good, with only one participant withdrawing from the study, prior to the post-intervention assessment sessions, due to unavailability.

Taken together, these results suggest that the intervention improved episodic memory by increasing the amount of accurate information retrieved and produced transfer to two complex cognitive tasks: problem-solving and divergent creative thinking.

### A process-based intervention

Our starting hypothesis was that our intervention should act as a *process-based* intervention as opposed to a *strategy-based* one. As previously mentioned, a *strategy-based* intervention involves learning how and when to apply new or alternative methods for performing a specific cognitive task. This approach compensates for impaired processes by leveraging those that remain intact (e.g., compensate for retrieval deficit by relying on encoding strategies), placing substantial reliance on metacognitive capacities. On the other hand, a *process-based* intervention involves improving a particular cognitive ability through repetitive engagement in tasks designed to target underlying core processes (e.g., episodic retrieval and scene construction) in a cognitively demanding manner, with the aim of restoring their functioning. Regarding the ESI training program, we mentioned that no encoding or retrieval strategies were taught during the intervention, and that metacognitive awareness was not encouraged. The intervention is therefore not thought to rely on the conscious engagement of encoding or retrieval strategies. Instead, the focus of the intervention was on repeated practice of a procedure which is assumed to place participants in a retrieval mode, independently of their will and awareness. This retrieval mode is assumed to facilitate the retrieval of event-specific details and their subsequent recombination during the scene construction process, thus targeting and improving this core process. Given that scene construction underpins multiple cognitive tasks the intervention was expected to produce transfer to other complex cognitive functions. Consequently, our starting hypothesis was supported by the fact that our ESI training program possesses all the characteristics of a process-based intervention.

Taken together, our results are consistent with this hypothesis. If our intervention acted as a *strategy-based* intervention, transfer effects would have been limited to tasks that lend themselves to the conscious reuse of the technique as a strategy – that is, to the free recall task – where participants were explicitly asked to recall event-specific details. Furthermore, the strong transfer effects we observed on very different tasks supports the notion that core common processes were improved by the intervention and repeated ESI practice.

Overall, in addition to replicating the findings of previous ESI-related studies and strengthening the role of episodic retrieval in a variety of cognitive functions, our study strengthen the idea that it is possible to design process-based interventions that focus on episodic retrieval and have more potential to produce transfer to complex cognitive tasks than strategy-based interventions (Belleville et al., 2006, 2018; von Bastian et al., 2022; Zimmermann et al., 2016). Although our study fills a gap due to the paucity of studies supporting such an idea, further studies are required. Those will have to propose theories on what underlies the changes induced by such process-based interventions.

### Theoretical and clinical implications

The intervention proposed here and results obtained can be linked to the capacity-efficiency model (von Bastian & Oberauer, 2014; see also von Bastian et al., 2022). This model proposes that an intervention can induce transfer by expanding a particular cognitive resource – cognitive *capacity* – and/or increasing the *efficiency* with which this capacity is used or its underlying processes/subprocesses. For instance, in the context of episodic retrieval, gains in capacity would correspond to an increase in the amount of information retrieved, whereas gains in efficiency would correspond to the acquisition of retrieval strategies or routines, as well as the automatization of basic processes. Gains in capacity can therefore be underpinned by gains in efficiency. Some evidence suggests that the transfer produced for most of the intervention was driven by gains in efficiency.

Our findings suggest that the intervention may have induced gains in efficiency by increasing the level of automatization with which a specific retrieval orientation is adopted, which in turn increased the efficiency of a core scene/event construction process involved in a range of cognitive tasks, like the ones used in this study. This led to the formation of richer, more specific episodic representations, which supported participants’ performance in those tasks. Such a hypothesis could explain how the classic effects of the ESI technique, known to be transient, were enhanced and maintained by repeated practice of the technique during the intervention.

Our training based on ESI can be compared to the Memory Specificity Training (MEST) program (for a review, see Barry et al., 2019). In the MEST program, participants learn to focus on and pay attention to the details that make a memory specific and unique. Moreover, they are trained to detect when they shift towards more general or unspecific recall, with the aid of feedback and guidance. The MEST program has been associated with improvements in memory specificity and depressive symptoms. Its effect on emotional symptoms is interesting but unlike our intervention, the MEST program promotes metacognitive awareness and operates as a strategy-based intervention.

Developing cognitive interventions that produce transfer to a broad range of cognitive abilities may have major implications in promoting healthy aging. Indeed, these interventions could be used to simultaneously improve a set of cognitive abilities impacted by aging, even when these abilities are used in different contexts. They would thus be more efficient compared to interventions that only produce narrow benefits and teach strategies that can only be used in a very specific context. Episodic retrieval appears to play a crucial role in a broad range of cognitive abilities, and in the daily functioning of an individual. Our intervention was specifically designed to improve episodic retrieval and may therefore produce transfer to those abilities in older adults. Interventions like ours could help older adults adapt flexibly to changing contexts, contribute to their cognitive health and improve their quality of life. Such interventions may also have implications for other clinical populations with episodic retrieval difficulties, such as patients with depression and anxiety disorders (see Brown et al., 2014; Williams et al., 2007).

### Limitations

Results reported in the present study should be taken with caution. The study was not designed as an efficacy study and should not be interpreted as such: The absence of an active control group and the small sample size limits our conclusion to the feasibility of a multi-session memory intervention based on the ESI and its potential in producing transfer effects. The absence of a control group means that the potential influence of a test-retest effect on the results cannot be completely ruled out. However, the absence of post-intervention differences between the ESI and no-ESI conditions for free recall and problem solving, coupled with the absence of an induction order effect, minimizes the likelihood of such influence. In our future efficacy study (third part of the SPECTRA study), the intervention will be compared to an active control intervention using a larger sample size. This study did not include follow-up assessments aimed at investigating the long-term effects of the training. Additionally, the recruitment of only two male participants limited our ability to incorporate sex as a fixed effect in our analyses. To address this we are actively exploring varied outreach strategies to enhance the inclusion of more male participants in our future studies. Finally, as performance was not assessed during the training sessions, the analysis of its potential impact on the intervention effects is beyond the scope of our current study.

## Conclusion

This proof-of-concept study aimed to assess whether a training program consisting of repeated practice of the ESI technique can improves episodic retrieval and induces transfer to complex tasks in healthy older adults. We first replicated the induction effects typically observed on tasks that rely on episodic retrieval (free recall, problem-solving and creative divergent thinking) in a sample of older adults. We also observed intervention effects on nearest, near and far transfer outcome measures. The observed transfer effects are encouraging and suggest that the intervention may increase the efficiency of a common underlying core process and produce improvements that extend beyond the trained task. Finally, we observed a good adherence to the intervention. The next step will be to compare our intervention with an active control intervention on a larger sample of healthy older adults and investigate the cerebral changes induced by the intervention. Ultimately, this intervention may contribute to reducing age-related changes in complex cognitive functions and improve the quality of life of older adults.

## Data Accessibility Statement

The data that support the findings of this study and the Supplementary Material of this study are openly available in OSF (https://osf.io/) at http://doi.org/10.17605/OSF.IO/HT8DS.

## Additional File

The additional file for this article can be found as follows:

10.5334/joc.323.s1 Supplementary Material. This document provides details of the material used in the tasks.

## Footnotes

We preferred the term specific detail to internal detail used in the scoring manual since an internal detail is necessarily event-specific.

## Appendix

**Appendix A:** Model structures.

EFFECT	TASK	MODEL STRUCTURES

Induction	Free recall	specific_details ~ induction + (1 | participant_ID)

	Recognition	dprime ~ induction + (1 | participant_ID)

	MEPS	relevant_steps ~ induction + (1 | participant_ID)

	AUT	creativity_score ~ induction + (1 | participant_ID)

		categories_of_appropriate_uses ~ induction + (1 | participant_ID)

Intervention	Free recall	specific_details ~ induction*time + (1 | participant_ID)

	Recognition	dprime ~ induction+time + (1 | participant_ID)

	Associative recognition	dprime ~ distinctiveness+time + (1 | participant_ID)

	MEPS	relevant_steps ~ induction*time + (1 | participant_ID)

	AUT	creativity_score ~ induction+time + (1 | participant_ID)

		categories_of_appropriate_uses ~ induction*time + (1 | participant_ID)

MEPS: Means-End Problem Solving Task; AUT: Alternate Uses Task (divergent creative thinking.
