# Supplementary material for: The SPECTRA Study: Validating a New Memory Training Program based on the Episodic Specificity Induction to Promote Transfer in Older Adults
Source: J Cogn. 2023 Oct 6;6(1):57. doi: 10.5334/joc.323 (PMC10558027; doi:10.5334/joc.323)
Supplement: Supplementary Material. — This document provides details of the material used in the tasks. [file joc-6-1-323-s1.pdf]

Supplementary Material

Experimental settings

Computerized tasks were performed on an HP ProBook 440 G6 connected to a 20.3" external monitor (LG W2242TQ) with a 1680 x 1050 resolution and equipped with an external mouse and keyboard.

Free recall and recognition tasks

Video-clips – Forty 10-second royalty-free and audio-free video-clips were downloaded from the website Pexels (http://www.pexels.com). These video-clips depicted clearly identifiable real-word scenarios (e.g., "Two little girls playing chess"), encompassing a spectrum of characters including men, women, children, young adults, and older adults representing diverse ethnic backgrounds. The selection process was stringent, focusing on video clips set within indoor environments, which were rich in terms of objects, colours, furniture and action. Each videoclip was categorized into one of 10 distinct groupings, comprising four videos, based on the number of characters involved, and their respective ages and genders. Subsequently, four lists of 10 videoclips were created, each composed of one video-clip from each category. The emotional valence of the selected videos was either positive or neutral, but not negative.

Auditory and visual video-clip titles – For each video clip, a nine-syllable title was created in French that clearly identified the depicted event (e.g., "Two little girls playing chess"). An accompanying audio track was also created for each title, consisting of an

artificial but realistic female voice enunciating the title, using the website TTSMP3 (www.ttsmp3.com).

Auditory and visual video-clip statements – For each videoclip, six nine-syllable statements were generated about different aspects of the videoclip: Two were about visual aspects (e.g., the colour of an object), two were about spatial aspects (e.g., the location of an object), and two were about the actions carried out (e.g., who performed a given action). An audio track was also created for each statement, featuring the same artificial but realistic female voice enunciating the statement, using the website TTSMP3 (www.ttsmp3.com).

Associative recognition task

Characters—A total of 14 blocks (two practice blocks, 12 experimental blocks) of four characters were created using The Sims™ 4 video game. Half of the blocks were assigned to the "LOW DISTINCTIVENESS" context, where the characters in each block were designed to have a low visual distinctiveness from each other. Half of the blocks were assigned to the "HIGH DISTINCTIVENESS" context, where the characters within each block were designed to have a high visual distinctiveness from each other. The distinctiveness of the characters in each block was configured based on 11 dimensions: gender (man vs. woman), build (heavy vs. light), skin colour (blue, red, green, or yellow), age (child vs. adult), hair colour (blond, brown, ginger, or white), head covering (beanie, beret, cap, or hat), glasses (with vs. without), sleeve length (short vs. long), pant length (short vs. long), shoes (sneakers vs. flip-flops), and facial features.

Within each block in the LOW DISTINCTIVENESS context, the modalities of five dimensions (gender, build, skin colour, age, hair colour) were identical for the four characters of a given block. For the remaining six dimensions, the modalities were pseudo-randomized across the four characters in the block as follows: For a two-modality dimension, two characters shared the same modality, while for a four-modality dimension, one character was assigned to each modality.

Within each block in the HIGH DISTINCTIVENESS context, the modalities of all 11 dimensions were pseudo-randomized across the four characters within the block. In this context, only two (not four) modalities of the skin colour dimension were assigned in each block. The facial features of the characters in each block were very similar, but very different from those of the characters in the other blocks. To ensure utmost distinctiveness, no two characters shared the same modality configuration (out of the 260 possible configurations). Of the 14 blocks created, two blocks (one of each context) were selected for practice after changing their skin colour modality to black, white and brown. A screen capture of each character was taken.

Actions—Twelve different types of actions were selected (e.g., 1: sitting on, 1: playing) using The Sims™ 4 video game. For each action type, four corresponding objects on which to perform the action were selected (e.g., sitting on …1.1: a chair, 1.2: a stool, 1.3: a bench, 1.4: an armchair). Within each block of characters, the four characters performed the same action but on a different object. The association between actions and distinctiveness context (high vs. low) was counterbalanced between subjects across two lists (A vs. B). For instance, the "Sitting on__" action was presented in the LOW

DISTINCTIVENESS context for half of the participants, while this action was presented in the HIGH DISTINCTIVENESS context for the other half. Two additional action types and eight matching objects were included in the practice blocks.

Videos—Using The Sims™ 4 video game, 48 videos were generated and recorded for Action List A (one for each character), and 48 were recorded for Action List B. Eight additional videos were recorded for the practice blocks using the two additional action types and the eight matching objects added for these blocks. Each video started with a full shot of the motionless character presented in front of the camera for approximately one second. The character then moved toward the spectator in the direction of the object, which was not present in the frame at that time. The camera remained in front of the character and tracked its movement for approximately three seconds. The object appeared in the camera's range and to the right of the character when the character stopped moving. Finally, the character performed the action on the object for approximately three seconds. The scene took place in an empty room with grey walls and floor. On the floor, a path between two blue stripes was always visible and was followed by the character. The average length of the videos was 7.52 seconds (SD = 1.20).

Audio—For each object, an audio recording was created using Balabolka 2.11 in which an artificial but realistic female voice vocalized the type of action followed by the name of the object after a short pause (e.g., "Sitting on__a CHAIR"). Forty-eight audio tracks were recorded. Eight additional audio tracks were recorded for the practice blocks based

on the two additional action types and the eight matching objects included for these blocks.

Problem-solving task (MEPS)

Stories – Four lists of four problem stories were selected from the problem stories used by Madore & Schacter (2014). Each problem story contained a beginning problem and an ending solution. Half of the problem stories were standard MEPS stories (Platt & Spivack, 1975), each introducing the problem in third-person and involving different fictional characters. These standard MEPS stories contained problems such as finding a watch or making new friends. The other half of the problem stories were self-relevant MEPS stories created by Madore & Schacter (2014) using data collected from an independent sample of older adults that identified goals that they found personally relevant in their own lives (Spreng & Schacter, 2012). Each problem was introduced from the first-person standpoint, and included problems, such as enhancing exercise routines and adopting healthier dietary habits.

Divergent creative thinking task (AUT)

Objects – Twenty object cues were selected from Madore et al. (2016) and designated common, everyday objects (e.g., newspaper, eye glasses, umbrella) appearing in the official test booklet for the AUT (Guilford et al., 1960).

Induction

Induction videoclips – Ten one-minute video-clips depicting animated characters were downloaded from the website YouTube (www.youtube.com). For each videoclip, the scene took place in a single context. Videoclips were selected and controlled to depict an indoor environment, which was rich in terms of objects, colours, furniture and action.

Intervention videoclips – Sixteen one-minute video-clips were downloaded on the website YouTube (www.youtube.com). Each videoclip featured a complex scenario featuring the famous British comic character Mr. Bean performing common activities in a familiar location (e.g., ordering food in a restaurant). For each videoclip, the scene took place in a single context. Videoclips were selected and controlled to depict an indoor environment, which was rich in terms of objects, colours, furniture and action.

Induction scripts – The French version of the script of the standard version of the ESI technique (ESI condition) and what we call the “general thoughts interview” instead of the term “control induction” used by Madore et al. (2014) (No-ESI condition) – both designed to be used with videoclips – was derived from Purkart, Vallet, et al. (2019).
